# Supplementary material for: Thyroid hormone receptor α controls larval intestinal epithelial cell death by regulating the CDK1 pathway
Source: Commun Biol. 2022 Feb 7;5:112. doi: 10.1038/s42003-022-03061-0 (PMC8821549; doi:10.1038/s42003-022-03061-0)
Supplement: Supplementary file 2 — Supplementary Information [file 42003_2022_3061_MOESM2_ESM.pdf]

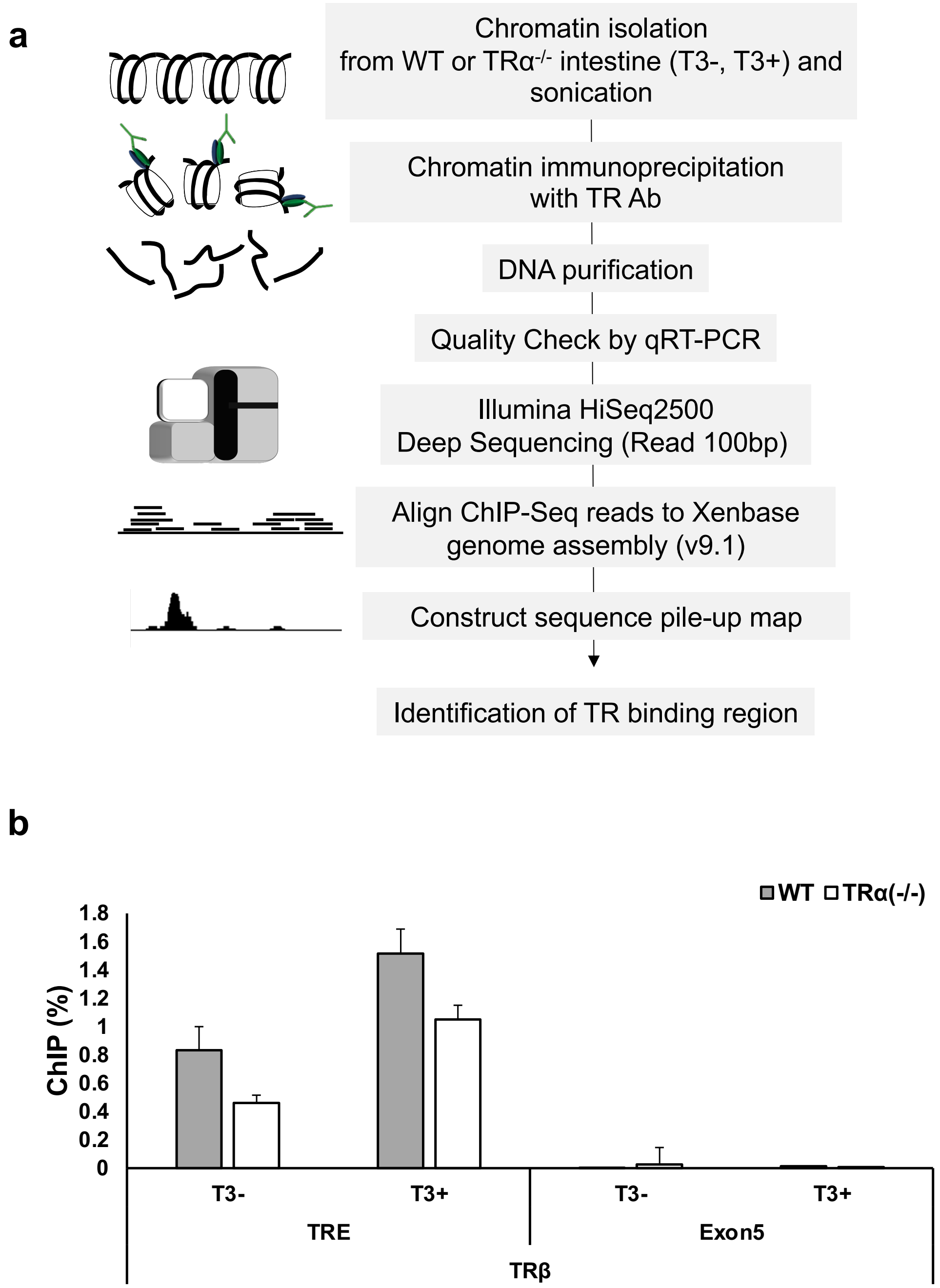

Fig. S1. Experimental design and quality check of the ChIP samples.

(a) Schematic diagram of the ChIP-Seq procedure. Wild type (WT) and homozygous TRα knockout (TR$\alpha^{-/-}$) tadpoles at stage 54 were treated without or with T3 for 18 hours. Chromatin was isolated from the intestine and sonicated with a Bioruptor. The chromatin fragments were cross-linked and immunoprecipitated by using a rabbit polyclonal TR antibody. After DNA purification, the quality of the ChIP DNA was checked by qPCR analysis for the well-known TRE region of TRβ gene (see B) before 100 bp paired-end sequencing with Illumina HiSeq2500 was performed. ChIP-Seq reads were aligned to *X. tropicalis* genome assembly (v9.1). Finally, the data were normalized by the base coverage profile of the pile-up and the peak sites were used for identification of the TR binding sites.

(b) Validation of the ChIP samples from the WT and TR$\alpha^{-/-}$ intestine. The ChIP samples in (a) was analyzed by PCR for the presence of the region containing the TRβ TRE or TRβ exon 5, the negative control region lacking a TRE.

<table>
<tr><th rowspan="2">Tissue</th><th rowspan="2">Genotype</th><th rowspan="2">T3</th><th rowspan="2">No</th><th rowspan="2">Total peaks</th><th rowspan="2">Common peaks</th><th colspan="3">Pileup</th><th rowspan="2">Mean height by base</th></tr>
<tr><th>max</th><th>min</th><th>mean</th></tr>
<tr><td rowspan="12">Intestine</td><td rowspan="6">WT</td><td rowspan="3">-</td><td>1</td><td>46608</td><td rowspan="3">11469</td><td>738</td><td>2</td><td>4.31</td><td>4.24</td></tr>
<tr><td>2</td><td>60134</td><td>1694</td><td>3</td><td>7.68</td><td>7.55</td></tr>
<tr><td>3</td><td>50207</td><td>2508</td><td>4</td><td>12.06</td><td>11.26</td></tr>
<tr><td rowspan="3">+</td><td>1</td><td>47106</td><td rowspan="3">17930</td><td>866</td><td>3</td><td>7.87</td><td>7.64</td></tr>
<tr><td>2</td><td>57418</td><td>1540</td><td>4</td><td>12.44</td><td>11.73</td></tr>
<tr><td>3</td><td>64193</td><td>1977</td><td>4</td><td>12.70</td><td>12.13</td></tr>
<tr><td rowspan="6">TRα$^{-/-}$</td><td rowspan="3">-</td><td>1</td><td>59774</td><td rowspan="3">9770</td><td>315</td><td>2</td><td>3.81</td><td>3.79</td></tr>
<tr><td>2</td><td>50729</td><td>498</td><td>3</td><td>6.73</td><td>6.63</td></tr>
<tr><td>3</td><td>54233</td><td>511</td><td>3</td><td>6.61</td><td>6.53</td></tr>
<tr><td rowspan="3">+</td><td>1</td><td>22150</td><td rowspan="3">8160</td><td>481</td><td>3</td><td>7.11</td><td>7.77</td></tr>
<tr><td>2</td><td>33834</td><td>665</td><td>3</td><td>7.51</td><td>3.91</td></tr>
<tr><td>3</td><td>42420</td><td>717</td><td>3</td><td>7.80</td><td>7.42</td></tr>
</table>

Fig. S2. Summary of the peak and pileup values for different ChIP-Seq samples. ChIP-Seq were performed for 3 technical replicates of the intestine samples from wild-type and TRα$^{-/-}$ tadpoles with or without 18 hour treatment with 10 nM T3. The ChIP-Seq FASTQ sequences were aligned to the *Xenbase* genome sequence to identify the peaks and determine the peak heights (Pileup, in base pairs). Note that the common peaks refers to peaks present in all three replicates of the samples.

Motif	Name	Sample	P-value	Target Sequences with Motif	% of Targets Sequences with Motif
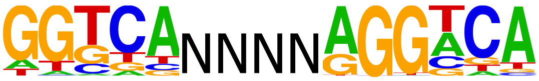	THRa(NR)	WT T3-	1.00E-97	6285	7.63%
		WT T3+	1.00E-153	7904	8.71%
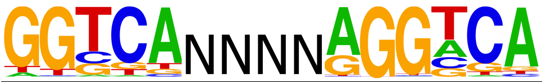	THRb(NR)	WT T3-	1.00E-60	7847	9.53%
		WT T3+	1.00E-87	9692	10.69%
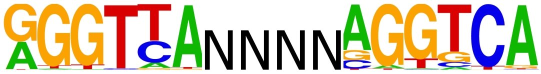	LXRE(NR) DR4	WT T3-	1.00E-12	1188	1.44%
		WT T3+	1.00E-35	1476	1.63%

Fig. S3. The DR4 TRE sequences are highly enriched in the TR-binding peaks detected by ChIP-Seq in the wild type intestine. Homer de novo motif search of all peak regions in the wild type intestine showed that THRa, THRb and LXRE (DR4) motifs were highly enriched. Note that the software used for the analysis did not show the first residue of the first repeat for THRa and THRb motifs. In any case, the result indicate clearly that DR4 TREs bound by TR have the consensus sequence of GGTCA and (A/G)GGTCA separated by 4 bases, basically identical to the idealized DR4 TRE made of AGGTCAnnnnAGGTCA.

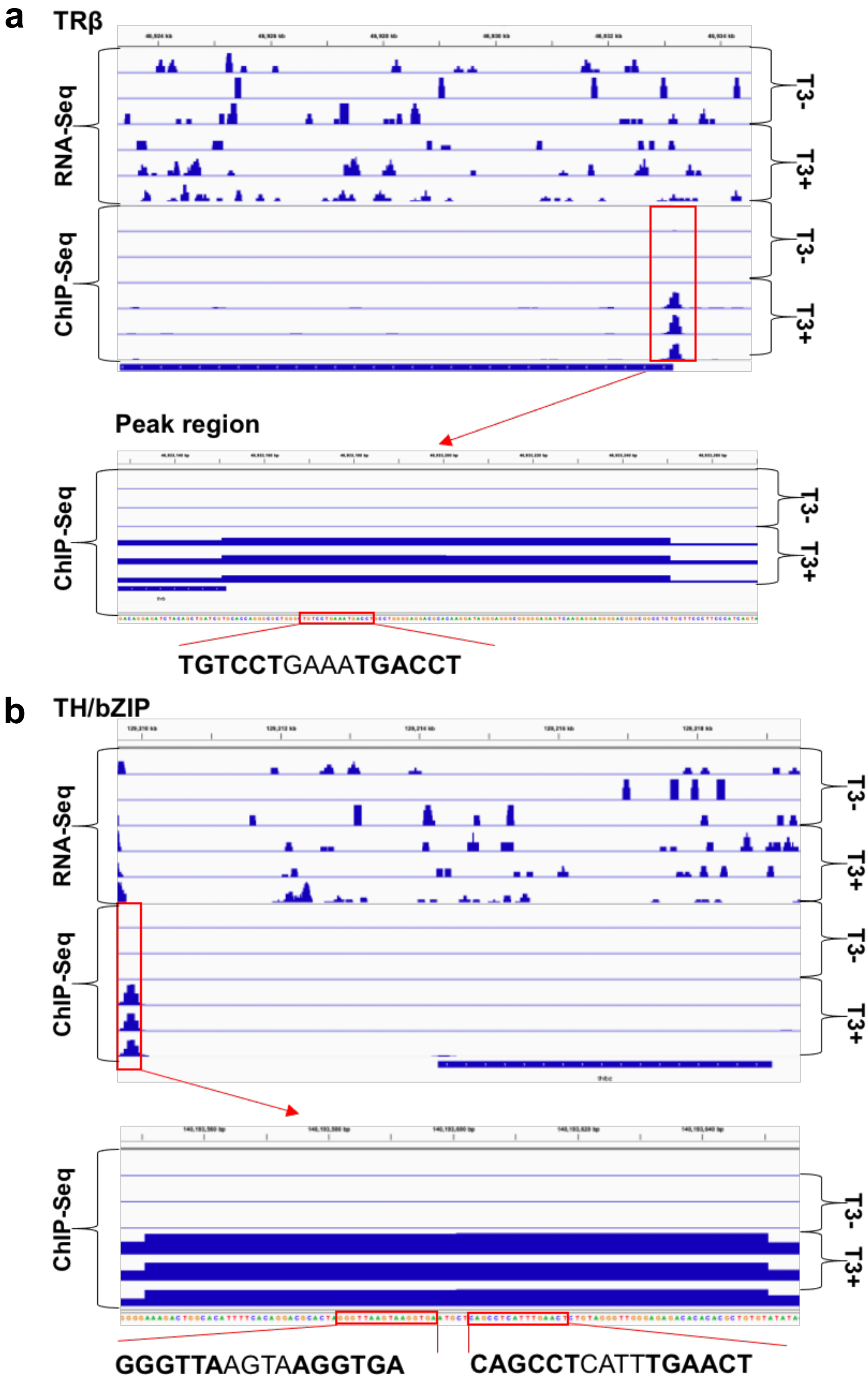

Figure S4. Representative views of ChIP-Seq and RNA-Seq data: (a) TRβ and (b) TH/bZip. Blue peaks in each wiggle plot represent the normalized ChIP-seq and RNA-seq reads, respectively. There were three technical replicates for each sample (+T3 or -T3). The red-boxed region in the ChIP-Seq plot was enlarged to show the presence of previously characterized TREs in the TRβ and TH/bZip genes, with the known TREs are shown in bold letters.

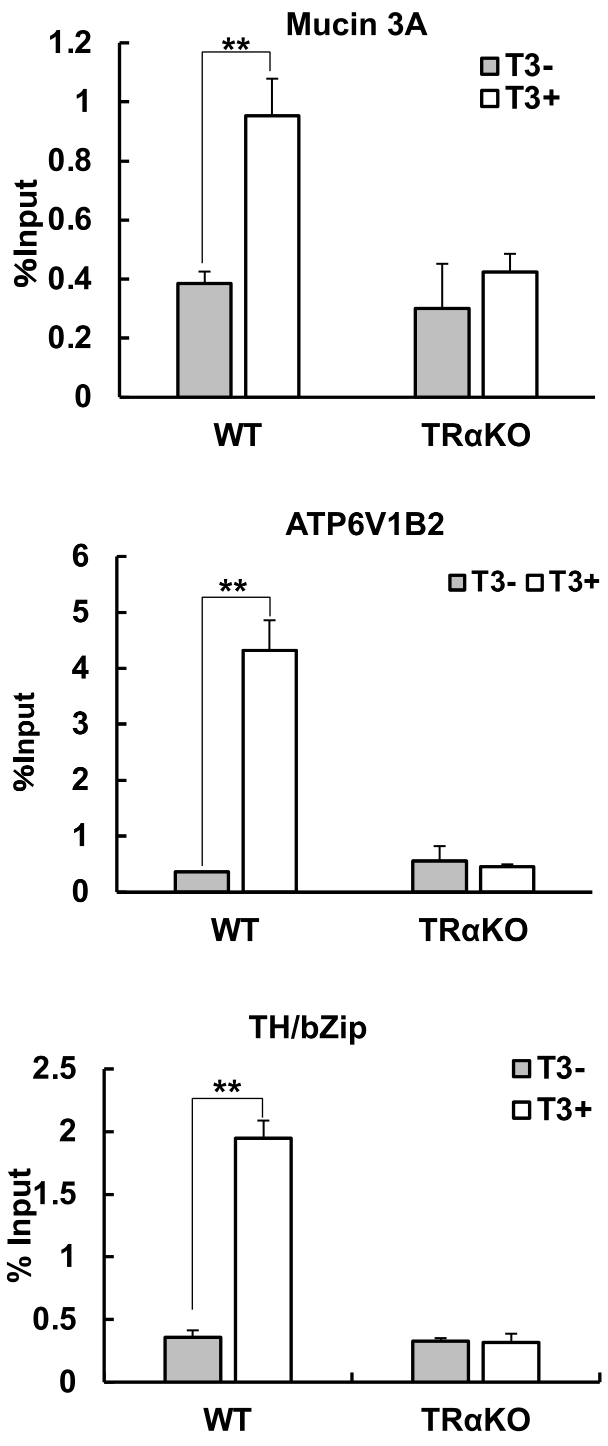

Fig. S5. TRα knockout reduces TR binding to the TRE region in the tadpole intestine. Five stage-matched wild type and TRα knockout tadpoles at stage 54 were treated with or without 10 nM T3 for 18 hr. The intestine was isolated for ChIP assay by using an anti-TR antibody. The immunoprecipitated DNA was analyzed by real-time PCR for the presence of the TRE region in Mucin3A, ATP6V1B2 and TH/bZip. Note that TR binding to the TRE region was significantly reduced in the intestine of TRα knockout tadpoles. Error bars indicate SEM. ** $p<0.01$.
