# Supplementary material for: Thyroid hormone receptor α controls larval intestinal epithelial cell death by regulating the CDK1 pathway
Source: Commun Biol. 2022 Feb 7;5:112. doi: 10.1038/s42003-022-03061-0 (PMC8821549; doi:10.1038/s42003-022-03061-0)
Supplement: Supplementary file 3 — Description of Additional Supplementary Files [file 42003_2022_3061_MOESM3_ESM.pdf]

Description of Additional Supplementary Data Files

File name: Supplementary data
Description:

Supplementary Data 1. ChIP-Seq data of wild-type intestine without T3 treatment
Supplementary Data 2. ChIP-Seq data of wild-type intestine with T3 treatment
Supplementary Data 3. List of genes bound by TR in wild-type intestine
Supplementary Data 4. Counts and positions of ChIP-Seq Peaks present in all 3 replicates of the indicated sample
Supplementary Data 5. List of GO terms enriched in genes with TR-binding detected in wild-type intestine
Supplementary Data 6. List of pathways enriched in genes with TR-binding detected in wild-type intestine
Supplementary Data 7. ChIP-Seq data of TRα-/- intestine without T3 treatment
Supplementary Data 8. ChIP-Seq data of TRα-/- intestine with T3 treatment
Supplementary Data 9. List of genes bound by TR in TRα-/- intestine
Supplementary Data 10. Summary of genes with TR-binding regions
Supplementary Data 11. List of GO terms enriched in genes with TR-binding detected in TRα (-/-) intestine
Supplementary Data 12. List of pathways enriched in genes with TR-binding detected in TRα (-/-) intestine
Supplementary Data 13. List of GO terms enriched among TR-bound genes in wild-type but not TRα (-/-) intestine
Supplementary Data 14. List of pathways enriched among TR-bound genes in wild-type but not TRα (-/-) intestine
Supplementary Data 15. List of TR-bound and T3 up-regulated genes in the "cell cycle" pathway
Supplementary Data 16. Primers and probes used for TaqMan-FAM based real time PCR
Supplementary Data 17. Primers used for ChIP-PCR
Supplementary Data 18. Figure 2a_Raw data. List of GO terms enriched in genes with TR-binding detected in wild-type intestine
Supplementary Data 19. Figure 2b_Raw data. List of pathways enriched in genes with TR-binding detected in wild-type intestine
Supplementary Data 20. Figure 6a_Raw data. List of pathways enriched among TR-bound genes in wild-type but not TRα (-/-) intestine
Supplementary Data 21. Figure 7b-c_Raw data. Body and Intestinal length after 2 days of T3 treatment
Supplementary Data 22. Figure 7d_Raw data. TUNEL positive area in the intestine after 2 days of T3 treatment
