# Supplementary material for: Thyroid hormone receptor α controls larval intestinal epithelial cell death by regulating the CDK1 pathway
Source: Commun Biol. 2022 Feb 7;5:112. doi: 10.1038/s42003-022-03061-0 (PMC8821549; doi:10.1038/s42003-022-03061-0)
Supplement: Supplementary file 5 — Reporting Summary [file 42003_2022_3061_MOESM5_ESM.pdf]

Corresponding author(s): Yun-Bo Shi

Last updated by author(s): Dec 28, 2021

Reporting Summary

Nature Portfolio wishes to improve the reproducibility of the work that we publish. This form provides structure for consistency and transparency in reporting. For further information on Nature Portfolio policies, see our Editorial Policies and the Editorial Policy Checklist.

Statistics

For all statistical analyses, confirm that the following items are present in the figure legend, table legend, main text, or Methods section.

n/a	Confirmed	
☐	☒	The exact sample size (*n*) for each experimental group/condition, given as a discrete number and unit of measurement
☐	☒	A statement on whether measurements were taken from distinct samples or whether the same sample was measured repeatedly
☐	☒	The statistical test(s) used AND whether they are one- or two-sided<br>*Only common tests should be described solely by name; describe more complex techniques in the Methods section.*
☐	☒	A description of all covariates tested
☐	☒	A description of any assumptions or corrections, such as tests of normality and adjustment for multiple comparisons
☐	☒	A full description of the statistical parameters including central tendency (e.g. means) or other basic estimates (e.g. regression coefficient) AND variation (e.g. standard deviation) or associated estimates of uncertainty (e.g. confidence intervals)
☒	☐	For null hypothesis testing, the test statistic (e.g. *F*, *t*, *r*) with confidence intervals, effect sizes, degrees of freedom and *P* value noted<br>*Give P values as exact values whenever suitable.*
☒	☐	For Bayesian analysis, information on the choice of priors and Markov chain Monte Carlo settings
☒	☐	For hierarchical and complex designs, identification of the appropriate level for tests and full reporting of outcomes
☒	☐	Estimates of effect sizes (e.g. Cohen's *d*, Pearson's *r*), indicating how they were calculated

Our web collection on statistics for biologists contains articles on many of the points above.

Software and code

Policy information about availability of computer code

Data collection	N/A
Data analysis	Statistical analyses were performed by using the Prism 8 statistics software (GraphPad Software Inc., San Diego, CA) and Statcel computer software (OMS, Ltd., Saitama, Japan). Raw sequencing data in FASTQ format were aligned to X. tropicalis genome assemblies (Ensembl Xenopus_tropicalis.JGI_4.2 and Xenbase v9.1) with Bowtie2 software (version 2-2.3.4.1) and redundant reads were removed from final bam files with samtools software (version 1.9). Peak enrichments were detected with MACS2 software (version 2.1.1.20160309). Pathway and gene ontology (GO) analyses with MetaCore software (GeneGo Inc., CA, USA), and used the KEGG Pathway database for visualization.

For manuscripts utilizing custom algorithms or software that are central to the research but not yet described in published literature, software must be made available to editors and reviewers. We strongly encourage code deposition in a community repository (e.g. GitHub). See the Nature Portfolio guidelines for submitting code & software for further information.

Data

Policy information about availability of data

All manuscripts must include a data availability statement. This statement should provide the following information, where applicable:

- Accession codes, unique identifiers, or web links for publicly available datasets
- A description of any restrictions on data availability
- For clinical datasets or third party data, please ensure that the statement adheres to our policy

Raw read datasets for all ChIP-seq samples are available under Gene Expression Omnibus (GEO) accession number GSM4913228 to GSM4913239.

March 2021

Field-specific reporting

Please select the one below that is the best fit for your research. If you are not sure, read the appropriate sections before making your selection.

☒ Life sciences ☐ Behavioural & social sciences ☐ Ecological, evolutionary & environmental sciences

For a reference copy of the document with all sections, see nature.com/documents/nr-reporting-summary-flat.pdf

Life sciences study design

All studies must disclose on these points even when the disclosure is negative.

Sample size	N/A. Many different tadpoles were pooled together, a standard approach in the field.
Data exclusions	None.
Replication	The ChIP-Seq was done with three technical replicates of samples from pooled tadpole tissues.
Randomization	The tadpoles were randomly selected based on stages.
Blinding	Animals were randomly grouped into two sets treated with or without thyroid hormone

Reporting for specific materials, systems and methods

We require information from authors about some types of materials, experimental systems and methods used in many studies. Here, indicate whether each material, system or method listed is relevant to your study. If you are not sure if a list item applies to your research, read the appropriate section before selecting a response.

Materials & experimental systems

n/a	Involved in the study
☐	☒ Antibodies
☒	☐ Eukaryotic cell lines
☒	☐ Palaeontology and archaeology
☐	☒ Animals and other organisms
☒	☐ Human research participants
☒	☐ Clinical data
☒	☐ Dual use research of concern

Methods

n/a	Involved in the study
☐	☒ ChIP-seq
☒	☐ Flow cytometry
☒	☐ MRI-based neuroimaging

Antibodies

Antibodies used	Anti-TR antibody
Validation	Previously characterized and published as referenced in the text.

Animals and other organisms

Policy information about studies involving animals; ARRIVE guidelines recommended for reporting animal research

Laboratory animals	Xenopus tropicalis
Wild animals	None
Field-collected samples	None
Ethics oversight	All Xenopus tropicalis experiments were approved by the Animal Use and Care Committee of Eunice Kennedy Shriver National Institute of Child Health and Human Development.

Note that full information on the approval of the study protocol must also be provided in the manuscript.

ChIP-seq

Data deposition

☒ Confirm that both raw and final processed data have been deposited in a public database such as GEO.

☒ Confirm that you have deposited or provided access to graph files (e.g. BED files) for the called peaks.

Data access links<br>*May remain private before publication.*	*For "Initial submission" or "Revised version" documents, provide reviewer access links. For your "Final submission" document, provide a link to the deposited data.*
Files in database submission	Gene Expression Omnibus (GEO) accession number GSM4913228 to GSM4913239
Genome browser session<br>(e.g. UCSC)	*Provide a link to an anonymized genome browser session for "Initial submission" and "Revised version" documents only, to enable peer review. Write "no longer applicable" for "Final submission" documents.*

Methodology

Replicates	Three technical replicates
Sequencing depth	Paired-end reads with length of 51 bp, total read pairs are 13 ~ 25 millions. Among them 1.3 ~ 7.6 millions are uniquely mapped.
Antibodies	Anti-TR antibody
Peak calling parameters	Mapping: bowtie2 -X 1000 --very-sensitive --no-mixed --no-unal -x Bowtie2_Genome_Files/Xenopus_tropicalis.Xenbase_v9.1<br>Peak calling: MACS2 callpeak, default parametes and without control
Data quality	fastqc was performed for raw and trimmed fastq records. Duplicated reads were removed from bam files. Total 6736 ~ 19664 peaks have enrichment fold >= 5 and FDR <= 0.05.
Software	Raw sequencing data in FASTQ format were aligned to X. tropicalis genome assemblies (Ensembl Xenopus_tropicalis.JGI_4.2 and Xenbase v9.1) with Bowtie2 software (version 2-2.3.4.1) and redundant reads were removed from final bam files with samtools software (version 1.9). Peak enrichments were detected with MACS2 software (version 2.1.1.20160309).
